# Behaviour Change for Physical Activity Is Feasible and Effective in Women Living with Metastatic Breast Cancer: A Pilot Two-Arm Randomised Trial

**DOI:** 10.3390/cancers18020338

**Published:** 2026-01-21

**Authors:** Mark Liu, Sharon Kilbreath, Jasmine Yee, Jane Beith, Elizabeth Dylke

**Affiliations:** 1 Faculty of Medicine and Health, The University of Sydney, Camperdown, NSW 2006, Australia; 2 Faculty of Health, Medicine and Behavioural Sciences, University of Queensland, Brisbane, QLD 4072, Australia; 3 Faculty of Science, The University of Sydney, Camperdown, NSW 2006, Australia; 4 Chris O’Brien Lifehouse, Camperdown, NSW 2050, Australia

**Keywords:** breast cancer, metastatic cancer, physical activity, behaviour change, quality of life, physical function

## Abstract

Background/Objectives: Physical activity benefits women with metastatic breast cancer. Past trials are typically well-resourced and supervised, but home-based interventions may be preferable and more accessible. This pilot trial evaluated the feasibility and preliminary efficacy of a remotely delivered behaviour change intervention aiming to increase physical activity for women with metastatic breast cancer. Methods: A 12-week, two-arm trial involved 20 women with metastatic breast cancer randomised 1:1 to a generic recommendation group or behaviour change group. Both groups received a physical activity recommendation, Fitbit^®^ watch, diary, and nine phone/video call sessions. The behaviour change group received individualised advice around physical activity benefits, motivation, barriers, and social support; the generic recommendation group completed a recurring symptom questionnaire. Feasibility outcomes were recruitment, retention and adherence rates. Acceptability was evaluated with a structured interview at trial completion. Preliminary efficacy outcomes included 5-day Actigraph wear, 6 min walk distance, 30 s sit-to-stands, and questionnaires for self-reported physical activity, quality-of-life, fatigue, behavioural factors, and patient-specific function. Results: Recruitment, retention, and adherence rates were 63% (n = 20/32), 80% (n = 16/20), and 76% (137/180 sessions), respectively. Participants across both groups reported that participation was acceptable, and their behaviour change was perceived as sustainable. Preliminary change scores for efficacy measures favoured the behaviour change group, except some quality-of-life and behavioural factor subscales. Conclusions: Participants were receptive to the trial, and feasibility and efficacy measures were positive. This indicates that a behaviour change intervention for unsupervised physical activity is acceptable and can be beneficial to women with metastatic breast cancer, warranting further exploration.

## Simple Summary

Physical activity can improve health and wellbeing for women living with metastatic breast cancer. Previous research has mostly been performed in supervised and well-resourced contexts. This study explored whether a home-based, remotely delivered program could support women with metastatic breast cancer to be more active. Twenty women took part in a 12-week programme and received an activity tracker, written educational materials, and regular phone/video calls from an exercise physiologist. One half received personalised support on motivation, barriers, and social support, while the other half received general advice only. We examined whether the programme was practical, acceptable, and showed signs of benefits. Most women completed the programme and reported that it was helpful and manageable in daily life, and women receiving personalised support tended to show greater improvements. These findings suggest that remotely delivered behaviour change programmes are a practical and beneficial way to support physical activity in this population.

## 1. Introduction

Women with breast cancer are routinely recommended to undertake physical activity for its far-reaching benefits, including improved physical function, quality of life, and fatigue [1]. Historically, there has been a strong bias in the research focusing on women with early-stage cancer, leaving a gap in knowledge about physical activity for women with metastatic breast cancer [2]. Women living with metastatic disease can often be assumed to be “palliative”, and inappropriate for supportive care such as exercise and physical activity programmes [3]. However, with medical advancements increasingly improving survival, many women with metastatic breast cancer may benefit from physical activity but have been excluded from exercise oncology research [4].

Of the few studies conducted for women with metastatic breast cancer, most interventions involved exercise undertaken in facilities with supervision [5]. However, when provided with an option of exercise location, women commonly preferred home-based settings due to factors of convenience, cost, and travel [6]; this was further confirmed in a European multinational survey [7]. Home-based unsupervised interventions can be effective [8] but require consideration as the reduced contact can be insufficient for impacting outcomes [9]. Applying a behaviour change approach to home-based interventions could, therefore, be advantageous for facilitating change in reduced-contact contexts. Behaviour change interventions aim to address the factors that impact health behaviours like physical activity, and have been applied successfully for early-stage breast cancer patients [10]. Frequently featured behaviour change techniques, such as goal-setting, education, monitoring and feedback, have also been shown to be relevant to those living with metastatic disease in qualitative studies [11,12]. However, whether they are sufficient for increasing physical activity in the metastatic cancer population is unknown.

Using individualised behavioural advice [13,14] to support women with metastatic breast cancer to reach the standard exercise oncology recommendation (150 min/week of moderate intensity aerobic activity) [15,16] has not been explored. Programme features evidenced to facilitate physical activity in other cancer services include remote delivery for increased reach and access [17], as well as self-monitoring to build towards self-management [18], which can be reinforced using wearable activity monitors [19]. If feasible, a programme with these elements would be an initial step towards developing a potentially cost-effective, implementable, and sustainable approach to facilitating physical activity for women with metastatic breast cancer [20].

This pilot trial, therefore, evaluated the feasibility and efficacy of an individualised behaviour change intervention for increasing physical activity for women with metastatic breast cancer. The primary feasibility outcomes were recruitment, retention, and adherence rates, as well as acceptability of participating in the trial. The secondary efficacy outcomes were physical activity, physical function, quality of life, fatigue, and behaviour change factors.

## 2. Materials and Methods

This report follows the CONSORT guidelines for pilot and feasibility trials (Supplementary Material S1) (Trial registration number: Australian New Zealand Clinical Trials Registry, Number ACTRN12620000743965, 16 July 2020) [21].

### 2.1. Design

A 12-week pilot randomised trial was conducted, allocating participants 1:1 to either of the following: (i) a generic recommendation group that received a general physical activity recommendation; (ii) a behaviour change group that received individualised behavioural advice. A two-arm design was applied to specifically evaluate the behaviour change techniques, with the generic recommendation group being akin to an attention control [22].

### 2.2. Participants

Recruitment spanned May 2022 to April 2023. Participants were approached during routine appointments at a metropolitan outpatient oncology clinic, or through emails distributed via a breast cancer consumer newsletter (Breast Cancer Network Australia).

Eligibility criteria were as follows: (i) women diagnosed with metastatic breast cancer, (ii) >18 years old, (iii) English-speaking, (iv) Eastern Cooperative Oncology Group Grade 0 to 2, i.e., ambulatory and capable of self-care, and (v) physically inactive, (i.e., self-reporting <150 min/week of moderate intensity physical activity). Women were excluded if they had a contraindication determined by their primary oncologist.

Prior to enrolment, informed consent was sought and a general pre-exercise screening tool [23] was administered to assess the risk of exercise-related adverse events; notable flags were discussed with their oncologist. Randomisation was performed using sealed, opaque envelopes prepared by an external researcher, which were opened by the assessing researcher after baseline assessments.

### 2.3. Intervention

This trial was informed by preceding qualitative studies conducted in collaboration with a consumer representative (Cancer Voices NSW) [11,12]. These perspectives were incorporated into the development of the study to enhance patient-centredness.

The trial schedule is presented in Table 1. Both groups received nine remotely delivered sessions (phone or video call) over 12 weeks with an exercise physiologist experienced in delivering behavioural advice for chronic condition management (ML): six weekly sessions, tapering to three fortnightly sessions. Both groups were given a physical activity diary and activity monitor (Fitbit^®^ Inspire) as optional self-monitoring tools; data from these were not collected. A general recommendation of 150 min/week of moderate intensity physical activity was given in the first session to both groups [15,16], and individualised goal-setting for frequency, duration, and intensity was discussed in the subsequent weeks. Participants were educated to self-monitor intensity using the activity monitor or the respiration rate “talk test”.

During their sessions, the generic recommendation group was asked to complete a physical wellbeing questionnaire (10-item subset of the European Organisation for Research and Treatment of Cancer Quality of Life Questionnaire [24]). This provided an opportunity for self-reflecting on potential benefits of physical activity and was not a part of the trial measures.

The behaviour change group received individualised behaviour change advice. The initial overview session was informed by previous qualitative research on being active while living with metastatic breast cancer [11,12]. Topics included the benefits of physical activity, goal-setting, time management, self-efficacy, social support, and using rewards (Supplementary Material S2). Subsequent sessions involved motivational interviewing [25], feedback, discussing barriers, and suggesting behaviour change techniques tailored to what was relevant to each participant [13].

### 2.4. Measurement

Baseline and 12-week assessments were conducted in-person, at a university research clinic or in the community at a location convenient for participants, e.g., their residence or a public park; this was replicated pre/post-intervention. Age, weight, height, time since initial and metastatic diagnoses, metastatic sites, treatments, and comorbidities were also collected. The researcher who completed the post-intervention measurements was blinded to group allocation. Questionnaires were completed again via email (Qualtrics XM) at 18 weeks.

#### 2.4.1. Primary: Feasibility Outcomes

The primary feasibility outcomes were the rates of recruitment (women enrolled/approached), retention (completion of 12- and 18-week assessments), adherence (attended/scheduled sessions), and physical adverse events [26]. The acceptability of participating was evaluated at 12 weeks through structured interviews and field notes (conducted by ED) to capture feedback on the following: (i) participation (assessments, sessions, resources), (ii) perceived sustainability of any behaviour changes, and (iii) suggestions for future research.

#### 2.4.2. Secondary: Efficacy Outcomes

Moderate-to-vigorous physical activity and steps were measured with a hip-worn activity monitor (Actigraph^®^ GT3X, Ametris, FL, USA). Participants were asked to wear the device for all waking hours for five consecutive days. Data processing was performed using ActiLife (v6.5.4). Moderate and vigorous intensity was calculated using the Freedson adult cut-points [27], and wear time was validated with Choi’s algorithm (≥10 h/day) [28,29].

Other secondary outcomes consisted of self-reported physical activity (International Physical Activity Questionnaire [30]); physical function (6 min walk test [31], 30 s sit-to-stand test [32], and the Patient-Specific Functional Scale [33]); quality of life and fatigue (European Organisation for Research and Treatment of Cancer Quality of Life Questionnaire—Core 30 [24] and Fatigue 12 [34]), and behavioural factors (Transtheoretical Model of Behaviour Change scales for Stage of Change (5-item), Self-Efficacy (18-item), Decisional Balance (10-item), Processes of Change (30-item) [35], and the Social Support for Exercise Scale (13-item) [36]). The published questionnaires have demonstrated reliability and validity [24,34,36] and were scored following the original algorithms.

### 2.5. Data Analysis

For the primary feasibility outcomes, percentage proportions were calculated for the recruitment, retention, and adherence rates, and responses to the structured interviews were summarised using conventional content analysis. Secondary efficacy outcomes were descriptively analysed as unadjusted means, from which change scores and between-group effect sizes were calculated (Cohen’s d: small = 0.2, medium = 0.5, large = 0.8). Data analyses were carried out on SPSS (v28, IBM, New York, NY, USA).

#### Sample Size

Ten participants per arm was the smallest sample able to inform a main trial with 90% power to detect a large (0.8) effect size, with two-sided 5% significance [37].

## 3. Results

Participant characteristics (n = 20) are shown in Table 2. Median participant age was 62 years, median time since metastatic disease diagnosis was four years, and the most common metastatic sites were bone, liver, and lung.

### 3.1. Recruitment, Retention and Adherence

**Figure 1:**
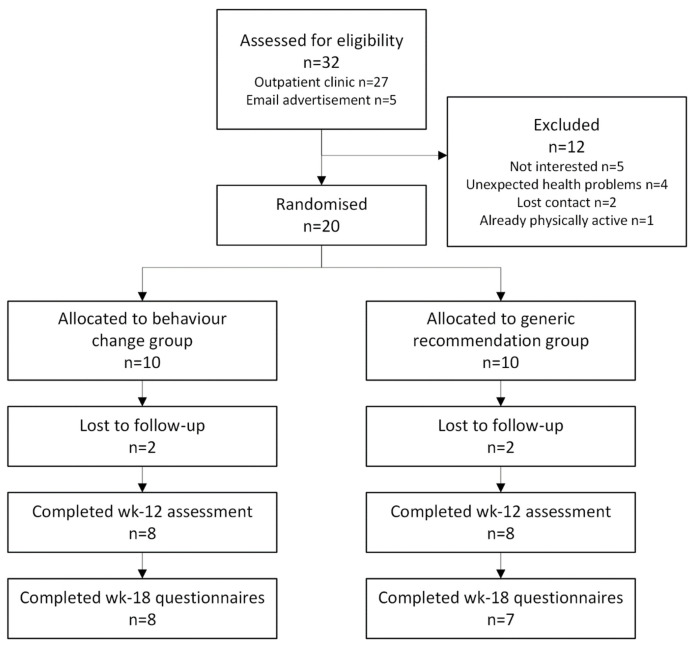
CONSORT flow diagram.

To recruit 20 participants, 32 women were approached, with 12 not interested, ineligible, or nonrespondent (recruitment rate: 63% over 12 months) (Figure 1). Four participants, two from each group, dropped out due to loss of contact (retention rate: 80%). One additional participant did not complete the 18-week follow-up. Participants attended 137/180 total sessions (76%) (137/163 and 84%, accounting for public holidays and clinic/researcher unavailability), with the behaviour change group adherence slightly higher (88%) than the generic recommendation group (80%). Participant reasons for missed sessions were as follows: forgetting (55%), feeling unwell (24%), IT problems (7%), holidays (7%), and work commitments (7%).

#### Adverse Events

Two participants reported a fall during incidental activity, neither of which resulted in injury.

### 3.2. Acceptability

#### 3.2.1. Trial Processes

All participants reported the recruitment, consenting, and communication processes as acceptable. Four participants opted to meet at their residence or a public park to perform the in-person assessments, and the remainder attended the university research clinic.

#### 3.2.2. Assessments

Participants reported mixed perspectives on the trial assessments. The broad scope of questionnaires was helpful for probing factors that may not have been previously considered. However, some participants found certain sections to be repetitive, or included some personally irrelevant questions, e.g., relating to family and significant others, or gyms and exercise equipment. Two participants with musculoskeletal considerations required accommodations to the walking and sit-to-stand measures, i.e., a walking aid and a chair with armrests, but were otherwise able to complete the assessments. Conversely, some participants with little to no physical restrictions found that the physical function tests were too easy. Two participants did not complete the Actigraph measure, finding it too burdensome.

#### 3.2.3. Sessions

Participants found that the remote delivery was sufficient for the intervention content, and the convenience facilitated attendance. The two modes for participating were equally preferred: ten participants preferred video call, nine preferred phone call, and one used both. Median session duration for the behaviour change group was 23 min (IQR: 20–28), and generic recommendation group was 17 min (IQR: 14–21). Participants found that the frequency, commencing weekly and tapering to fortnightly, was beneficial for iteratively increasing their self-efficacy. Information for reinforcing incidental physical activity (i.e., walking for commuting, strenuous routine activities) and self-monitoring aerobic intensity (i.e., low versus moderate) were found to be particularly useful. Some participants compared the tailored approach favourably against similar information that is provided online in a generic non-tailored format. In addition to delivering the trial content, participants reported that regular checkpoints provided external motivation and accountability. Participants that self-reported little to no behaviour change still found the newly gained information to be useful and it may be applied in the future.

#### 3.2.4. Resources (Diary and Fitbit^®^)

All but one of the participants used the optional physical activity diary and Fitbit^®^ (Fitbit Enterprise, San Francisco, CA, USA) activity monitor. Some women assimilated the self-monitoring processes with their personal organisational processes, such as using their existing personal diaries or fitness watches. Participants also diarised their energy levels relative to treatment cycles in order to preplan periods of low or high activity, as well as diet and sleep patterns, adjusting these as additional health behaviours. The activity monitor was reported to be useful for quantifying activity minutes and steps as a reference for goal-setting and self-monitoring. Two participants used the sleep-tracking function and found the metrics to be insightful.

#### 3.2.5. Sustainability

The majority of participants that self-reported increased physical activity anticipated being able to sustain the change. The main contributing factors were having newfound confidence and self-belief, as well as wanting to maintain the benefits experienced, including general physical and psychosocial wellbeing, and specifically, the perceived impact on disease adverse effects by alleviating symptoms and improving treatment tolerance. Two participants reported little or no belief in sustaining any changes in behaviour, due to busyness with work, health-related barriers, or already being active to a personally adequate level.

#### 3.2.6. Other Feedback

Several participants reported desiring additional related services or information outside of physical activity, e.g., exercise physiology or physiotherapy services (particularly regarding bone metastases), resistance training, dietary advice, and social support networks. Some participants also suggested that an extended duration and tapering of the trial may be beneficial, e.g., monthly sessions after the 12-week point.

### 3.3. Efficacy Findings

Table 3 presents the changes and between-group differences for the objective outcome measures from baseline to week 12. Improvements in 6 min walk distance and sit-to-stands in 30 s were observed in both groups, favouring the behaviour change group, but neither group significantly increased their objective physical activity. As physical decline is typically expected with this clinical population, the behaviour change group may be viewed to have achieved a greater protective effect against this decline. Table 4 presents the changes and between-group difference for the questionnaire outcome measures from baseline to week 12 and 18. These favoured the behaviour change group for most outcome measures at both timepoints, except for some quality-of-life and behavioural factor subscales.

Using 6 min walk distance data, a subsequent primary trial with a desired power of 0.9 (α = 0.05) would require a sample size of 62 participants per group to detect a clinically significant difference of 25 m between groups [38], based on the variability of data (Shapiro–Wilk test *p* = 0.59) and 20% dropout rate.

#### Patient-Specific Functional Scale Activities

Participants identified a diverse range of activities for the Patient-Specific Functional Scale, from basic or instrumental activities of daily living to leisure exercise activities or general health and wellbeing. Basic activities of daily living included lower body function (traversing stairs, steps, and inclines), mobility (transitioning up and down from the floor, reaching to put on shoes and socks), getting dressed, and gripping. Instrumental activities of daily living included strenuous housework, shopping, driving, and gardening (for one rural participant, this involved farm and animal care). Leisure activities included dance, hiking, yoga, cycling, and snorkelling. The remaining activities were related to general health and wellbeing (aerobic activity, lifting weights, and sleep). For one participant with brain metastases, this included cognitive function such as memory recall and computer work.

## 4. Discussion

This trial demonstrated the feasibility and potential effectiveness of a behaviour change intervention for increasing physical activity in women with metastatic breast cancer. The feasibility outcomes were positive, and the trial was largely acceptable and perceived to be sustainable. Preliminary efficacy findings were promising and warrant a larger powered trial.

A pragmatic strength of this trial was the broad range of participants’ health characteristics afforded by the broad eligibility criteria. Exercise oncology trials commonly employ numerous exclusion criteria around specific disease sequalae and/or comorbidities [5]. This prioritises safety, which is required for trials involving novel, high-risk interventions or samples. However, generally increasing physical activity, as in this trial, should be considered low-to-negligible risk if the disease and symptoms are reasonably stable, and participants are screened for exercise contraindications. The current trial enrolled participants with many varying comorbidities, which demonstrates that such interventions are feasible for women with diverse clinical presentations, rather than only those with few or no comorbidities. Notably, the participant with the lowest baseline 6 min walk distance, at 234 m, was able to improve to 378 m despite being >75 years old, with bone and lung metastases, on intravenous chemotherapy, and using a walking aid.

This trial achieved solid rates of recruitment, retention, and adherence, given the protocol and clinical population. A systematic review analysed recruitment rates in trials that evaluated similar interventions for people with advanced cancer [39]. Across the included studies, mean recruitment rate was 49%, which positions this trial, at 63%, favourably. This is despite the eligibility criteria for this current trial being relatively simple and inclusive to reflect the broader population, which likely increased the rate denominator. However, comparing such trials may be confounded by differences in the target population. For example, it would be inappropriate to compare a trial recruiting relatively healthy men with advanced prostate cancer (recruitment rate 74%) [40] to a trial that recruited from palliative care programmes (recruitment rate 15%) [41]. Concurrently, many trial features were intended to increase the convenience of participating, such as the remote format, the proximity between the recruiting hospital and university research clinic, and the option for the assessments to be conducted in the community. Such aspects likely facilitated the retention rate of 80% compared to the mean rate of 76% of the studies included in the aforementioned review [39]. The adherence rate was also high, comparable to a similar remotely delivered physical activity trial for people with early-stage cancer [25]. Notably, recruitment commenced shortly after a COVID-19 lockdown when hygiene practices were still prevalent, particularly for clinical groups such as people with cancer. This further supports the feasibility of this trial, along with participants’ desire for such services, with the remote format well suited to mitigate infection risk.

Participants’ perspectives shared during the acceptability interviews provide insights for informing future research in this field. Although the behavioural advice was individualised, many participants suggested that a more multi-faceted intervention would increase personal relevance. The primary physical activity mode was walking, so that participants of any baseline ability could engage. Expectedly, participants suggested other physical activity types and supplementary supports according to their physical ability and personal interests. These included classes for exercise modes (e.g., yoga or Pilates), advice for related health behaviours (e.g., diet and sleep), and cancer-specific social support networks. The activities identified in the Patient-Specific Functional Scale highlight the diversity of needs and preferences in this cohort, with goals ranging from basic mobility to specific exercise hobbies. One participant specifically requested to be referred to the exercise physiology services at the recruiting hospital; similar avenues could be embedded into future trials. A higher level of intervention tailoring has been successfully applied for women with metastatic breast cancer in a goal-setting physical therapy programme in a single-arm feasibility study [42]. To accommodate for individuals’ heterogenous needs, a stepped care model could be well suited for future programmes, where participants receive tiered-level services and supports according to their needs [43].

The appropriateness of a stepped care model is also supported by observations within the generic recommendation group and discontinued participants. The improvements observed in the generic recommendation group show that a lower level of facilitation may still be effective for some women. While no behavioural advice was given, participants did inherently receive some behavioural facilitation from the accountability of attending scheduled sessions, which may be sufficient for motivated participants. Interestingly, participants that dropped out from this current study due to loss of contact performed relatively higher on the baseline functional assessments. It is plausible that they perceived they were already adequately active and physically well, leading to a lack of interest and hence their discontinuation. The addition of a more advanced or supervised component may have been more appropriate and engaging to such participants.

The efficacy findings should be considered within the context of the sample characteristics. Actigraphs are widely used in physical activity research, but the findings from this study suggest that they may not be the most appropriate measurement tool for this population. Firstly, two participants found the 5-day wear too burdensome and opted out of the measure. Secondly, wear time periods were not timed relative to participants’ treatment cycles, meaning external confounds may have impacted data collection between pre/post-intervention periods. Nevertheless, self-reported physical activity increased, and most other study outcomes saw clinically significant increases. Relative to other physical activity studies involving advanced cancer populations [38], change scores were clinically meaningful for 6 min walk distance (≥25 m), sit-to-stands in 30 s (≥2 repetitions), and the quality-of-life and fatigue questionnaires (≥5 points). Another factor to consider is the relatively high baseline physical capacity, with a sample median of 529 m for the 6 min walk test versus the normative 477 m across all breast cancer survivors [44]. A higher baseline can cause a ceiling effect that dampens the ability to improve, but positive changes were still observed. These findings underscore the notion that women with metastatic breast cancer can achieve and maintain a robust physical condition, and that they may experience benefits from increasing physical activity.

This trial raised some feasibility considerations for future research. Firstly, relating to the behavioural factor questionnaires, these scales are a useful diagnostic tool, but there is limited longitudinal evidence that these measures are correlated to changes in the behaviour, due to the numerous possible confounding factors [45]. Secondly, due to ongoing changing disease circumstances, it may be difficult to fully reach “maintenance” stage for their physical activity behaviour. If such an intervention was implemented into practice, ongoing (e.g., monthly) sessions may be beneficial.

## 5. Conclusions

In conclusion, this trial presents a feasible and potentially effective protocol for promoting physical activity in women with metastatic breast cancer. Participants found the trial to be acceptable and expressed diverse and insightful views on which aspects of the trial were helpful and what could be improved. Future research could explore, using a stepped care model, where participants are allocated an intervention most relevant to their health status, needs, preferences, and behavioural readiness.

## Figures and Tables

**Table 1:** Trial schedule.

Week	0	1	2–6	7–12	13	18
Type (Frequency)	Baseline Assessments	Initial Education (Single)	Introducing the Behaviour (Weekly)	Embedding the Behaviour (Fortnightly)	Post-Trial Assessments	Follow-Up Assessments
**Delivery**	In-person	Video or phone call			In-person	Remote, email
**Generic recommendation group**	Physical activity: 5-day Actigraph wear, IPAQ Physical function: 6MWT, 30s STS, PSFS Health factors: EORTC QLQ-C30, FA12 Behavioural factors: Stage of Change, Self-Efficacy, Decisional Balance, Processes of Change, Social Support for Exercise	Physical activity recommendation (150 min/week of moderate intensity) and parameters for goal-setting and self-monitoring (frequency, duration, and intensity).	Iterative, collaborative goal-setting and progression/regression. Physical wellbeing questionnaire.	Iterative, participant-led goal-setting and progression/regression. Physical wellbeing questionnaire.	Physical activity: 5-day Actigraph wear, IPAQ Physical function: 6MWT, 30s STS, PSFS Health factors: EORTC QLQ-C30, FA12 Behavioural factors: Stage of Change, Self-Efficacy, Decisional Balance, Processes of Change, Social Support for Exercise	Physical activity: IPAQ Physical function: PSFS Health factors: EORTC QLQ-C30, FA12 Behavioural factors: Stage of Change, Self-Efficacy, Decisional Balance, Processes of Change, Social Support for Exercise
**Behaviour change group**		Physical activity recommendation (150 min/week of moderate intensity) and parameters for goal-setting and self-monitoring (frequency, duration, and intensity). Overview of general behavioural advice (physical activity benefits, motivations, barriers and social support).	Iterative, collaborative goal-setting and progression/regression. Motivational interviewing and feedback on progress. Discussion of barriers and suggestions for behaviour change techniques.	Iterative, participant-led goal-setting and progression/regression. Transition to self-management by encouraging self-reflection on progress and potential future barriers. Discussion and planning for physical activity beyond study timeframe.		
**Assessment of feasibility**	Rates of recruitment, retention and adherence.				Acceptability: Structured interview	

IPAQ: International Physical Activity Questionnaire, 6MWT: 6 min walk test, 30s STS: 30 s sit-to-stand test, PSFS: Patient-Specific Functional Scale, EORTC: European Organisation for Research and Treatment of Cancer, QLQ-C30: Quality of Life Questionnaire—Core 30, FA12: Fatigue 12.

**Table 2:** Participant characteristics.

Characteristic, Median (IQR)	All (n = 20)	Behaviour Change Group (n = 10)	Generic Recommendation Group (n = 10)
Age, years	62 (60–66)	63 (59–76)	61 (60–64)
BMI, kg/m^2^	26 (24–28)	26 (24–27)	24 (23–26)
Years since metastatic diagnosis	4.0 (1.3–5.0)	2.0 (1.0–5.0)	4.0 (3.6–4.3)
Years since initial diagnosis	12 (8–14)	9 (5–14)	13 (11–14)
**ECOG Grade ^1^**			
0	8	4	4
1	10	5	5
2	2	1	1
**Metastatic sites**			
Bone, lumbar/hip/femoral	8	5	3
Liver	7	4	3
Lung	5	2	3
Other (e.g., cranial, lymphatic)	4	2	2
**Current treatment(s) ^2^**			
Chemotherapy	10	5	5
Hormone therapy	7	3	4
Targeted therapy	7	5	2
**Comorbidities ^2^**			
Musculoskeletal	10	6	4
Cardiovascular	6	3	3
Gastrointestinal	5	3	2
Endocrine/Thyroid	3	2	1
Anaemia	1	1	0
Lower limb oedema	1	1	0
Psychological	1	0	1
Respiratory	1	0	1
Surgery in the past 6 months	1	1	0
**Baseline objective measures, ** **median (IQR)**			
Actigraph MVPA minutes	61 (38–76)	58 (43–66)	65 (37–95)
Actigraph step count	5685 (3461–6888)	5311 (3612–6313)	6061 (4200–7124)
6 min walk distance (m)	529 (426–577)	515 (444–583)	544 (375–573)
Sit-to-stands in 30s	11 (8–13)	11 (9–13)	11 (8–14)

MVPA: moderate-to-vigorous physical activity. ^1^ Eastern Cooperative Oncology Group Performance Status, 0: No disease-related restriction, 1: Restricted in physically strenuous activity but ambulatory and able to carry out work of a light or sedentary nature, 2: Ambulatory and capable of all self-care but unable to carry out any work activities; up and about more than 50% of waking hours. ^2^ Participants could report multiple treatments and comorbidities.

**Table 3:** Change in objective outcome measures from baseline to week 12.

	Behaviour Change Group Mean (SD)	Generic Recommendation Group Mean (SD)	Between-Group Difference (95% CI)	Effect Size (Cohen’s d)
Actigraph MVPA minutes	−2 (16)	−31 (17)	29 (2 to 55)	1.72
Actigraph step count	108 (1254)	−1284 (1778)	1392 (164 to 4619)	0.90
6 min walk distance (m)	48 (43)	16 (55)	32 (−37 to 102)	0.66
Sit-to-stands in 30s	2 (4)	−1 (3)	3 (−2 to 8)	0.90

MVPA: moderate-to-vigorous physical activity.

**Table 4:** Change in questionnaire outcome measures, from baseline to week 12 and 18.

	Baseline to 12 Weeks				Baseline to 18 Weeks			
	Behaviour Change Group Mean (SD)	Generic Recommendation Group Mean (SD)	Between-Group Difference (95% CI)	Effect Size (Cohen’s d)	Behaviour Change Group Mean (SD)	Generic Recommendation Group Mean (SD)	Between-Group Difference (95% CI)	Effect Size (Cohen’s d)
IPAQ (MET minutes/week)	755 (683)	217 (1463)	538 (−1118 to 2195)	0.47	749 (1829)	−191 (1828)	940 (−4622 to 6502)	**0.51**
Patient-Specific Functional Scale (0–10)	2.0 (1.0)	−0.9 (1.0)	2.9 (2.1 to 3.8)	**2.83**	1.7 (1.5)	−1.2 (1.5)	2.8 (1.5 to 4.1)	**1.89**
EORTC QLQ−Core 30 (0–100)								
*Summary sco* *re*	7 (19)	5 (7)	2 (−17 to 21)	0.14	11 (21)	5 (7)	6 (−19 to 30)	0.38
*Global health status*	15 (38)	3 (7)	12 (−24 to 47)	0.43	19 (45)	6 (5)	14 (−66 to 94)	0.43
*Physical functioning*	−3 (11)	8 (11)	−10 (26 to 6)	−**0.90**	1 (5)	9 (10)	−8 (−35 to 19)	**−0.97**
*Role functioning*	6 (20)	14 (16)	−8 (−33 to 17)	−0.42	3 (27)	28 (10)	−25 (−78 to 28)	**−1.25**
*Emotional functioning*	4 (39)	10 (15)	−6 (−45 to 34)	−0.19	7 (48)	3 (5)	4 (−90 to 89)	0.12
*Cognitive functioning*	15 (26)	3 (7)	12 (−13 to 36)	**0.62**	25 (29)	0 (0)	25 (−27 to 77)	**1.20**
*Social functioning*	10 (22)	6 (9)	5 (−17 to 27)	0.29	14 (13)	0 (17)	14 (−33 to 61)	**0.94**
EORTC QLQ−Fatigue 12 (0–100) ^1^								
*Physical fatigue*	−18 (30)	−16 (15)	−3 (−34 to 29)	−0.12	−22 (35)	−13 (0)	−9 (−46 to 28)	−0.35
*Emotional fatigue*	−13 (30)	−7 (9)	−5 (−34 to 24)	−0.23	−19 (36)	−7 (6)	−11 (−50 to 28)	−0.43
*Cognitive fatigue*	−6 (15)	−3 (7)	−3 (−19 to 12)	−0.29	−19 (25)	−6 (10)	−14 (−43 to 16)	−**0.75**
*Interference with daily life*	−4 (33)	0 (30)	−4 (−48 to 39)	−0.13	−11 (34)	0 (33)	−11 (−72 to 50)	−0.33
*Social sequelae*	−25 (39)	−22 (27)	−3 (−48 to 43)	−0.08	−28 (39)	−11 (19)	−17 (−67 to 33)	**−0.54**
Behavioural factor scales (1–5)								
*Stage of Change* ^2^	0.8 (1.4)	0.5 (0.5)	0.3 (−1.1 to 1.6)	0.24	0.5 (1.5)	0 (2.0)	0.5 (−2.9 to 3.9)	0.28
*Self-Efficacy*	−0.1 (0.7)	0.8 (0.7)	−0.9 (−1.9 to 0.1)	**−1.22**	−0.3 (0.9)	0.7 (0.1)	−1.0 (−2.0 to 0)	**−1.49**
*Decisional Balance*	0.1 (0.4)	0.5 (1.3)	−0.4 (−1.8 to 1.0)	−0.44	−0.1 (0.4)	0.2 (0.3)	−0.3 (−1.0 to 0.3)	**−0.90**
*Processes of Change*	0.1 (0.4)	0.7 (1.4)	−0.6 (−2.2 to 0.9)	**−0.60**	0.2 (0.3)	0 (0.2)	0.1 (−0.3 to 0.5)	**0.51**
Social Support for Exercise (8–104)								
*Family*	5 (17)	2 (3)	4 (−28 to 36)	0.30	8 (8)	−1 (2)	9 (10 to 27)	**1.46**
*Friends*	−7 (15)	−2 (4)	−4 (27 to 18)	−0.38	−6 (18)	−6 (5)	−1 (−36 to 35)	−0.05

IPAQ: International Physical Activity Questionnaire, MET: Metabolic equivalent of task, EORTC QLQ: European Organisation for Research and Treatment for Cancer Quality of Life Questionnaire. **Bold** indicates Cohen’s d is >0.5 or <−0.5. ^1^ A lower score represents less symptomatology or problems. ^2^ Ordinal categories of 1 = Precontemplation, 2 = Contemplation, 3 = Preparation, 4 = Action, 5 = Maintenance.

## Data Availability

The data presented in this study are available on request from the corresponding author due to reasons of sensitivity.

## Supplementary Materials

The following supporting information can be downloaded at https://www.mdpi.com/article/10.3390/cancers18020338/s1. Supplementary Material S1: CONSORT extension Pilot and Feasibility Trials Checklist; Supplementary Material S2: Behaviour change group booklet.
