# Supplementary material for: Behaviour Change for Physical Activity Is Feasible and Effective in Women Living with Metastatic Breast Cancer: A Pilot Two-Arm Randomised Trial
Source: Cancers (Basel). 2026 Jan 21;18(2):338. doi: 10.3390/cancers18020338 (PMC12839420; doi:10.3390/cancers18020338)
Supplement: Supplementary file 1 [file cancers-18-00338-s001.zip › Supplementary Material 2. Behaviour change group booklet.pdf]

Supplementary Material 2. Behaviour change group booklet

Physical Activity for People Living with Cancer

Breast Cancer and Lymphedema Research Group
UNIVERSITY OF SYDNEY

Introduction

Regardless of age, health, or fitness level, essentially all people are likely to benefit from being more active. As a society, we are becoming less physically active, with life's **busy-ness** and **distractions** getting in the way of enjoying the simple things like spending more time outdoors and focusing on our health.

Simply **walking more** is perhaps the easiest and most effective way of living a healthier lifestyle. It can be done anywhere and anytime, it costs nothing, and it can be a good break in your day to either spend some time with people or spend time being alone. Going for a brisk walk a day can improve physical fitness and your overall quality of life.

This booklet provides some practical advice on being more active. The aim is for you to think of some personal goals to guide you to a healthier lifestyle. When living with cancer, although daunting, research has shown that being physically active is completely safe and can make a positive change in your life.

Benefits of physical activity

Doctors in the past would advise cancer patients to avoid exerting themselves and to rest. However, over the last few years, research has increasingly shown that it is better to be more active. Knowing the benefits can motivate you to start being physically active yourself. For example, regular activity such as walking can help:

- ✓ Reduce fatigue: people going through cancer regularly feel lethargic and have low energy, being regularly active can reduce this and improve fitness and energy levels.
- ✓ Reduce stress and anxiety: being physically active is one of the best ways to control stress by regulating hormones that improve mood.
- ✓ Improve your confidence: as you set and complete goals for yourself, this will boost your sense of control over your health.
- ✓ There are many other benefits, which include**:**
    - Weight management
    - Heart health
    - Mental clarity
    - Sleep quality
    - Socialising, if you enjoy walking with others

The first step

Whether this is your first time trying to be more active, or you are trying to restart old habits, it may not be a straightforward path. It is important to think about the "**why**" to get you started and motivate you to keep going towards a healthier lifestyle.

Think about and write down your top reasons why you want to be more active. For example, "being more active can give me energy to enjoy spending time with people important to me", or "being more active will make me feel better and more confident about my health".

1. ____

   ____

2. ____

   ____

3. ____

   ____

How to get started?

Being active **doesn't** mean spending money on exercise equipment or gym memberships, or having to change your daily routines to fit in exhausting sessions. Regular walking as part of your daily life is all you need to take advantage of the benefits of physical activity.

Here are some common worries and ways to get around them:

- <u>I don't have enough time…</u>
    - Think about it like other daily tasks you do to look after yourself, like brushing your teeth.
    - Have a set time, instead of trying to figure out if it will fit in around other things.
    - Include it as part of other routines you do throughout the day: when driving or taking public transport, leave some distance so you can walk the rest, or go for a walk when catching up with a friend instead of sitting at a café.

I'm not fit enough…

- Any increase in physical activity is beneficial to your health, don't worry if it doesn't seem like as much as you would like.
- You can start with just 5 to 10 minutes at a slow pace, and gradually build up your time and speed.
- It is common to not meet your goals straight away at start, learn to be patient and accept that there might be setbacks along the way.

I wouldn't have the discipline…

- The hardest part is getting started! It will get easier as you go.
- **Get others involved**. Tell people about your goals so they can remind and encourage you, or even join you in trying to be more active.
- Be flexible with your goals at the start and build up a more regular routine as you get more confident.
- Remember that everyone's journey to achieving their goals will look different. Be proud of yourself when you do well and celebrate by rewarding yourself.

How much should you do?

To get the best results, research recommends doing 150 minutes a week of physical activity at a moderate intensity.

How long?

This may sound like a lot, but it can be split up to fit your preference or fitness level. For example, you could do **30 minutes a day for 5 days a week**. Or, instead of 30 minutes straight, you could do three 10-minute blocks spread throughout the day.

How hard?

It is also important to pay attention to how hard you are working when you walk. You should notice your body warming up, your heart rate getting faster and your breathing getting deeper, but not too much where you can't talk naturally. This is considered to be "**moderate-intensity**" activity. Try walking briskly as if you are running late, or walking in areas with hills. Activity that is less intense is considered "**light-intensity**", which is still beneficial to your health but not as potent.

But most of all:

Although these are the recommended guidelines, it is most important to make sure your goals are **right for you**. Think about what you can do now and set small and reachable goals to get you started. Your body will adapt, and your confidence will increase as you go.

It is also important to **listen to your body** at every stage. Contact your family doctor or your breast cancer nurse if you ever feel dizzy, light-headed, discomfort or pain.

Here are some tips to make sure you feel good when being active:

- Make sure you have comfortable and supportive footwear.
- When going for longer walks, build up gradually at the start to warm up and slow down at the end to cool down.
- Find times during the day you feel the least tired.
- Drink plenty of water throughout the day.
- If you are feeling low on energy, try eating a healthy snack before your walk.

Activity Diary:

The tables below are for you to keep track of your activity during this study. Each day, write down the:

- How many minutes
- What intensity: light or moderate
- Notes: e.g. how you were feeling that day, reasons why you were or were not active

Week 1: Date: ____/____/____ to ____/____/____

Goal: to be active _____ times this week, for _____ minutes, at ___________ intensity

	Minutes:	Intensity:	Notes:
Day 1:		☐ Light<br>☐ Moderate	
Day 2:		☐ Light<br>☐ Moderate	
Day 3:		☐ Light<br>☐ Moderate	
Day 4:		☐ Light<br>☐ Moderate	
Day 5:		☐ Light<br>☐ Moderate	
Day 6:		☐ Light<br>☐ Moderate	
Day 7:		☐ Light<br>☐ Moderate	
**Weekly Total:**			

Online resources:

For more information on physical activity and cancer, visit:

- Breast Cancer Network Australia:

www.bcna.org.au/health-wellbeing/physical-wellbeing/exercise-and-staying-fit/

- Cancer Council:

www.cancercouncil.com.au/cancer-information/exercise-cancer/

- Cancer Australia:

lifestylerisk.canceraustralia.gov.au/info/exercise
